# When Do Hedonic and Eudaimonic Orientations Lead to Happiness? Moderating Effects of Orientation Priority

**DOI:** 10.3390/ijerph18189798

**Published:** 2021-09-17

**Authors:** Hezhi Chen, Zhijia Zeng

**Affiliations:** 1 Department of Psychology, School of Education, Zhejiang International Studies University, Hangzhou 310023, China; chenhezhi@zisu.edu.cn; 2 Mental Health Education Center, Zhejiang University of Finance and Economics, Hangzhou 310018, China

**Keywords:** orientations to happiness, orientation priority, psychological well-being, positive affect, negative affect

## Abstract

The effects of hedonic and eudaimonic orientations on individual well-being have received much scholarly attention. However, the empirical findings from previous research are not consistent, raising the question of when the pursuit of hedonia and eudaimonia lead to actual improvements in individual well-being. We argue that the relationship between orientations to happiness and well-being outcomes are moderated by orientation priorities, which reflect the relative level of importance individuals place on eudaimonic motives compared to hedonic motives. A total of 312 Chinese undergraduate students completed surveys assessing hedonic and eudaimonic orientations, orientation priorities, and well-being outcomes, including psychological well-being, positive affect, and negative affect. The results revealed that a eudaimonic orientation was positively related to psychological well-being, a hedonic orientation was positively related to positive affect, and both relationships were moderated by orientation priorities. For individuals who prioritized eudaimonia over hedonia, both orientations improved well-being. For individuals who prioritized hedonia over eudaimonia, the benefits related to well-being from both orientations decreased or disappeared. These findings suggest that orientation priorities are of equal importance in regard to hedonic and eudaimonic orientations.

## 1. Introduction

Orientations to happiness represent the values, motives, and goals of individuals that guide their behaviors to achieve happiness [1]. Hedonia and eudaimonia have been recognized as the two most prominent views of happiness [2]. Specifically, the former refers to seeking pleasure and comfort, whereas the latter refers to seeking personal growth and a meaningful life. Although philosophers have debated the types of happiness people should pursue since the Ancient Greek period, recent empirical research has focused on how the pursuit of hedonia and eudaimonia may affect individual well-being [1]. However, previous studies have reported inconsistent results, with some research showing that both hedonic and eudaimonic orientations promote well-being [3,4], whereas others have found that pursuing hedonia does not bring happiness and can even be harmful [5,6]. These conflicting findings suggest that the beneficial effects of hedonic and eudaimonic orientations are not straightforward and raise the question of when orientations to happiness improve individual well-being.

Previous studies have attempted to investigate this question. For instance, researchers divided the hedonic orientation into the pleasure orientation and the relaxation orientation and found that the pleasure orientation was generally beneficial to individual well-being, whereas the relaxation orientation had a neutral or even detrimental effect [7,8]. However, other studies suggested that the types of hedonic orientation are indistinguishable from one another [5,9]. Culture may also be a factor in determining the way in which pursuing hedonia and eudaimonia affects individual well-being. Specifically, a hedonic orientation was found to have a stronger positive relation with happiness in more individualistic cultures [10]. However, the role of culture cannot explain the inconsistent results regarding the relation between orientations to happiness and individual well-being within both collectivistic [5,11,12,13] and individualistic cultures [3,4,6,14]. Thus, the potential boundary conditions for when orientations to happiness lead to benefits in well-being require further exploration.

In the present study, we propose that the priority individuals place on a eudaimonic orientation relative to hedonic orientation may be a crucial moderator, and that pursuing both hedonia and eudaimonia will improve well-being only for those who prioritize eudaimonia over hedonia. Below, we elaborate on our hypotheses and provide supportive empirical evidence.

### 1.1. Orientations to Happiness and Well-Being

Several early works on orientations to happiness reported that both hedonic and eudaimonic orientations are beneficial to individual well-being. In a pioneering study, Peterson, Park, and Seligman [4] found that pleasure, engagement, and meaning orientations all positively predicted life satisfaction, and individuals who scored simultaneously low on all three orientations experienced the lowest levels of life satisfaction. These findings have been replicated with samples from various countries, including Australia, France, and China [11,15,16]. Similarly, using a different measure of orientations to happiness, Huta and Ryan [3] found that hedonic and eudaimonic orientations had distinct effects on well-being. In particular, a hedonic orientation was related more to positive affect, whereas a eudaimonic orientation was related more to a sense of meaning in life. Huta [1] concluded that hedonic and eudaimonic orientations were related to different aspects of well-being, as they promoted hedonic and eudaimonic behaviors, respectively, and thus a combination of hedonic and eudaimonic orientations would lead to the greatest well-being.

In addition, a hedonic orientation may be beneficial to eudaimonic well-being through an indirect path and vice versa. According to the broaden-and-build theory of positive emotions, positive emotions expand an individual’s immediate thought-action repertoire [17]; thus, people are more likely to engage in eudaimonic activities when they are in positive affective states [18]. Furthermore, lasting personal resources, ranging from physical and intellectual resources to social and psychological resources, can be obtained through pursuing eudaimonia. These resources can help individuals to cope with stress more efficiently and increase their resilience when facing adverse events, thereby improving their hedonic well-being [19,20].

In contrast, the hedonic adaptation hypothesis posits that trying to increase one’s positive affect as a focal goal may be ineffective [21]. This is because people are likely to become habituated to positive events; thus, positive emotions generated by these changes will become less frequent and intense over time [22]. In addition, when positive changes become the new norm, people will increase their aspirations for even more positive experiences to regain their initial feelings of happiness [21].

Empirical research has provided supporting evidence for the hedonic adaptation hypothesis. For example, Sheldon, Corcoran, and Prentice [6] demonstrated that striving to improve eudaimonic well-being did indeed lead to beneficial outcomes, whereas motivation to improve hedonic well-being did not affect longitudinal subjective well-being. Similarly, another study found that a eudaimonic orientation, but not a hedonic orientation, was related to affective experience and flourishing [23]. Further, in another longitudinal study, eudaimonic orientation was found to have positive effects on positive affect and life satisfaction after two months, whereas no effects were observed between hedonic orientation and subjective well-being components [12]. Moreover, daily eudaimonic behaviors (e.g., expressing gratitude for something someone did) were found to enhance positive emotions, one’s sense of meaning in life, and life satisfaction; in contrast, daily hedonic behaviors (e.g., buying new jewelry or electronics equipment just for oneself) did not affect the above happiness indicators [24].

Furthermore, a hedonic orientation may even be detrimental to individual well-being. A strong hedonic orientation has been found to be associated with low self-control, and thus might lead to dysfunctional behaviors and hinder people from achieving long-term goals [13,25]. For example, high levels of hedonic orientation were related with low levels of grit and investment in learning, which thereby led to poor academic achievement [14,26,27]. A hedonic orientation was also related with dysfunctional coping strategies (e.g., addictive behavior), which, in turn, further harmed well-being [7,28].

### 1.2. Orientation Priority as a Moderator

Combining the theories and empirical findings mentioned above, it can be suggested that the pursuit of hedonia can either facilitate or interfere with the pursuit of eudaimonia, which further determines whether hedonic and eudaimonic orientations are beneficial to individual well-being. Thus, the question remains as to when the pursuit of hedonia will be an obstacle in the pursuit of eudaimonia. One point which has been much neglected in previous research is that conflicts between hedonic and eudaimonic orientations in daily activities are common. For example, when presented with an opportunity to make a donation, hedonic motives will push people toward keeping the money for their own use, whereas eudaimonic motives will push them toward giving the money to support others’ welfare. Similarly, when confronted with a stressful challenge, hedonic motives will encourage people to procrastinate for temporary relief, whereas eudaimonic motives will urge them to make a consistent effort to stay on task. In such situations with conflicting motives, people will exert more effort to pursue the goals they consider more important [29]. Consequently, individuals decide their behaviors based on not only either a hedonic or eudaimonic orientation but also the relative importance of the two goals [30].

In the current study, we define orientation priority as the relative importance an individual places on a eudaimonic orientation over a hedonic orientation, especially when these two orientations are in conflict. When facing conflicting motives, for individuals who prioritize eudaimonia over hedonia, the eudaimonic orientation takes the dominant position in deciding their behavior, thus promoting long-term goal achievement and further improving well-being. However, the hedonic orientation promotes hedonic behavior in situations without motive conflicts, and thus can also increase well-being. For individuals prioritizing hedonia over eudaimonia, the hedonic orientation is the primary source affecting decision-making in situations with motive conflicts. In this case, the hedonic orientation may cause dysfunctional behaviors which hinder people from achieving long-term goals, thereby harming their well-being. In addition, as a eudaimonic orientation has less of an effect on decision-making, its positive effects on well-being may also decrease or disappear.

### 1.3. The Current Study

The aim of this study was to investigate when hedonic and eudaimonic orientations are beneficial to one’s well-being and when they are not. For this purpose, cross-sectional data on hedonic and eudaimonic orientations, orientation priorities, and well-being outcomes were collected. We posited that the relationships between orientations to happiness and well-being outcomes are moderated by orientation priorities. As such, we hypothesized the following:

**Hypothesis** **1** **(H1).** *Orientation priorities moderate the relationship between a hedonic orientation and well-being outcomes. For participants who prioritize eudaimonia over hedonia, a hedonic orientation will be positively related with well-being; for participants who prioritize hedonia over eudaimonia, a hedonic orientation will be negatively related with well-being.*

**Hypothesis** **2** **(H2).** *Orientation priorities moderate the relationship between a eudaimonic orientation and well-being outcomes. For participants who prioritize eudaimonia over hedonia, a eudaimonic orientation will be positively related with well-being; for participants who prioritize hedonia over eudaimonia, a eudaimonic orientation will not be related with well-being.*

## 2. Materials and Methods

### 2.1. Sample and Procedures

We conducted a cross-sectional survey with a convenience sample of 323 undergraduate students from one university in Hangzhou, China. The participants completed an online survey in the classroom for extra course credit. Nine participants failed in attention-check items and were removed from the analyses, which left a final sample of 314 participants (mean age = 18.53 years, standard deviation = 0.85; 148 male, 166 female).

### 2.2. Measures

Measures of hedonic and eudaimonic orientations and well-being outcomes were translated from English to Chinese and back to English by two bilingual colleagues, respectively. The two authors compared the original and back-translated versions, and non-equivalent translations were further modified after discussion. We performed confirmatory factor analysis (CFA) and calculated both Cronbach’s α and McDonald’s ω to ensure the validity and reliability of the measures used in the present study.

#### 2.2.1. Hedonic and Eudaimonic Orientations

Hedonic and eudaimonic orientations were measured using the Hedonic and Eudaimonic Motives for Activities (Revised) (HEMA-R) Scale developed by Huta [1]. This scale comprises five items to assess the hedonic orientation (e.g., “seeking pleasure”) and five items to assess the eudaimonic orientation (e.g., “seeking to use the best in yourself”). Respondents reported the degree to which they used each motive when engaging in their daily activities, using a seven-point Likert scale ranging from 1 (not at all) to 7 (very much). The scale has been found to have good psychometric properties in Chinese samples [9]. In the present study, the two-factor model had an adequate fit index (χ^2^ (29) = 84.52, χ^2^/df = 2.92, the comparative fit index (CFI) = 0.95, the Tucker-Lewis index (TLI) = 0.93, the root mean square error of approximation (RMSEA) = 0.078, the standardized root mean squared residual (SRMR) = 0.080). Cronbach’s α was 0.84 and 0.81, and McDonald’s ω was 0.85 and 0.81 for hedonic and eudaimonic orientations, respectively.

#### 2.2.2. Orientation Priorities

Based on previous research [31], we modified the HEMA-R Scale to assess orientation priorities using a pairwise comparison approach. Each of the five hedonic motives were compared with each of the five eudaimonic motives, which constituted 25 pairwise comparisons in total (e.g., “seeking pleasure—seeking to use the best in yourself”). Each type of motive appeared approximately equally on the left or right side of the comparison. Participants indicated the degree to which they prioritized one goal over the other in their daily activities on a seven-point Likert scale ranging from −3 (the left goal is much more important) to +3 (the right goal is much more important). Items with the eudaimonic motivation on the left side were reverse-coded. Higher scores indicated a higher prioritization of the eudaimonic orientation over the hedonic orientation. Considering the small sample size relative to the large number of items, we performed both parcel-level and item-level confirmatory factor analysis (CFA), in line with previous studies [32,33]. Each eudaimonic motive appeared in five items, and these five items generally showed high correlations. Thus, we grouped each of the five items into two parcels (one parcel with two items and the other with three items), which resulted in a total of ten parcels. The one-factor model with ten parcels showed an appropriate fit index (χ^2^ (31) = 97.83, χ^2^/df = 3.16, CFI = 0.97, TLI = 0.96, RMSEA = 0.083, SRMR = 0.047). For the item-level CFA, the model showed a less good but minimally acceptable fit index (χ^2^ (208) = 654.16, χ^2^/df = 3.15, CFI = 0.92, TLI = 0.88, RMSEA = 0.083, SRMR = 0.075). The factor loadings of all items were above 0.4. Cronbach’s α for this scale was 0.94 and McDonald’s ω was 0.94.

#### 2.2.3. Well-Being Outcomes

Similar to previous research [23], three different well-being indicators were used: psychological well-being, positive affect, and negative affect. Psychological well-being reflects eudaimonic experiences and functioning, and was measured using the Flourishing Scale [34]. This scale comprises eight statements (e.g., “I lead a purposeful and meaningful life”). Items are rated on a seven-point Likert scale ranging from 1 (strongly disagree) to 7 (strongly agree). This scale has been validated with Chinese samples in previous studies [35]. In the present study, the one-factor model showed an appropriate fit index (χ^2^ (17) = 48.38, χ^2^/df = 2.85, CFI = 0.96, TLI = 0.94, RMSEA = 0.077, SRMR = 0.042). Cronbach’s α was 0.84 and McDonald’s ω was 0.85.

Positive affect and negative affect reflect hedonic experience, and were measured with the Scale of Positive and Negative Experience [34]. The scale comprises six items related to positive experiences (e.g., “pleasant”) and six to negative experiences (e.g., “unpleasant”). Participants reported the frequency of each feeling over the past four weeks. Items were rated on a five-point Likert scale ranging from 1 (very rarely or never) to 5 (very often or always). The scale has shown good reliability and validity in Chinese samples [36]. In the present study, the two-factor model showed a good fit index (χ^2^ (50) = 102.79, χ^2^/df = 2.06, CFI = 0.98, TLI = 0.97, RMSEA = 0.058, SRMR = 0.046). Cronbach’s α was 0.92 and 0.88, and McDonald’s ω was 0.92 and 0.89 for positive affect and negative affect, respectively.

### 2.3. Statistical Analysis

We performed all tests using SPSS 23.0 (IBM, New York, NY, USA) software. First, we calculated the descriptive statistics and correlations of study variables. More importantly, we tested the hypothesized moderation effects of orientation priority on the relationship between hedonic and eudaimonic orientations and well-being outcomes using the PROCESS macro for SPSS (Model 1) [37,38].

## 3. Results

### 3.1. Descriptive Analyses

Table 1 shows the means, standard deviations, and zero-order correlations of the study variables. Orientation priority was positively related with the eudaimonic orientation (*r* = 0.48, *p* < 0.001) and negatively related with the hedonic orientation (*r* = −0.34, *p* < 0.001). Neither age nor gender was significantly correlated with any of the study variables; therefore, they were excluded from further analysis.

### 3.2. Moderation Analyses

To test whether orientation priorities played a moderating role in the relationship between hedonic and eudaimonic orientations and well-being outcomes, we conducted a series of multiple regression analyses. In these analyses, all variables were standardized before being entered into the model. Hedonic orientation, eudaimonic orientation, and orientation priority values were entered as Step 1, followed by the interaction between hedonic orientation and orientation priority and between eudaimonic orientation and orientation priority values in Step 2.

We first examined the link between hedonic and eudaimonic orientations and psychological well-being, and whether orientation priorities moderated this relationship (Table 2). For the main effects in Step 1, a eudaimonic orientation was positively related with psychological well-being (*ß* = 0.17, 95% CI (.03, 0.32), *p* = 0.018). Orientation priorities were also positively related with psychological well-being (*ß* = 0.25, 95% CI (0.10, 0.39), *p* = 0.001). A hedonic orientation was not related with psychological well-being (*ß* = 0.10, 95% CI (−0.04, 0.23), *p* = 0.160). In Step 2, the eudaimonic orientation × orientation priority interaction was statistically significant (*ß* = 0.15, 95% CI (0.05, 0.26), *p* = 0.003). However, the interaction between a hedonic orientation and orientation priorities was not statistically significant (*ß* = 0.06, 95% CI (−0.14, 0.17), *p* = 0.248).

**Figure 1:**
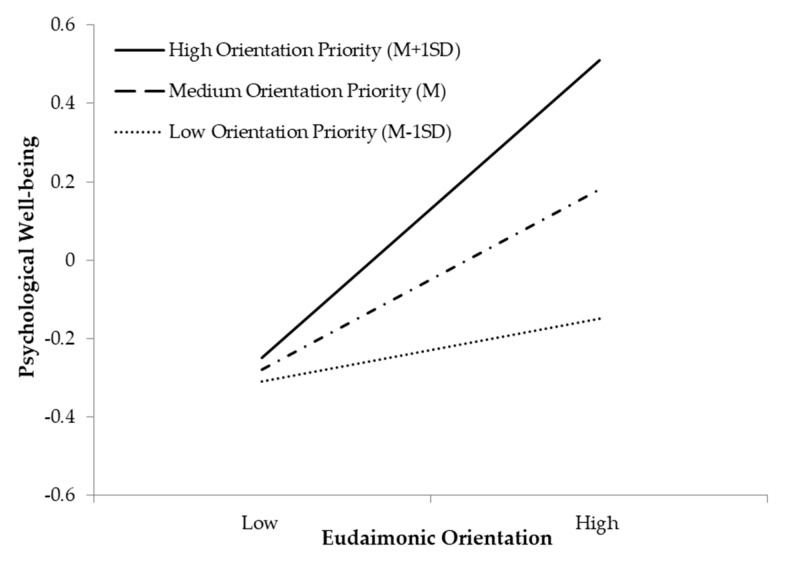
Orientation priorities moderates the effect of a eudaimonic orientation on psychological well-being.

Simple slope analyses revealed that for participants with a high orientation priority, a eudaimonic orientation was positively related with psychological well-being (*ß* = 0.39, 95% CI (0.19, 0.58), *p* < 0.001) (Figure 1). For participants with a medium orientation priority, a eudaimonic orientation had a relatively smaller but significant effect on psychological well-being (*ß* = 0.23, 95% CI (0.09, 0.38), *p* = 0.002). For participants with a low orientation priority, the relationship between eudaimonic orientation and psychological well-being was not significant (*ß* = 0.08, 95% CI (−0.08, 0.24), *p* = 0.339).

Next, we examined the link between hedonic and eudaimonic orientations and positive affect and the moderating effect of orientation priorities (Table 3). For the main effects in Step 1, a hedonic orientation was positively related with positive affect (*ß* = 0.14, 95% CI (0.00, 0.28), *p* = 0.046). Orientation priorities were also positively related with positive affect (*ß* = 0.16, 95% CI (0.01, 0.31), *p* = 0.039); however, a eudaimonic orientation was not related with positive affect (*ß* = 0.05, 95% CI (−0.10, 0.20), *p* = 0.483). In Step 2, the hedonic orientation × orientation priority interaction was statistically significant (*ß* = 0.12, 95% CI (0.01, 0.24), *p* = 0.033), whereas the interaction between eudaimonic orientation and orientation priority was not (*ß* = 0.08, 95% CI (−0.03, 0.18), *p* = 0.167).

**Figure 2:**
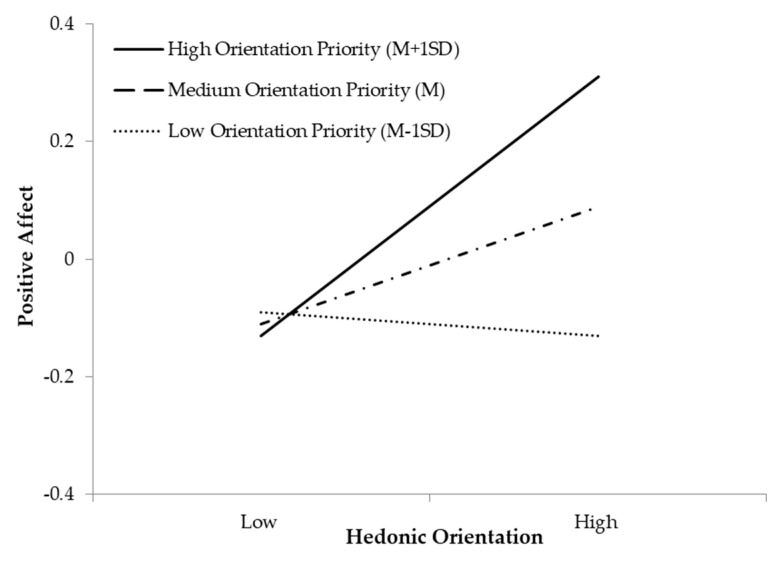
Orientation priorities moderate the effect of a hedonic orientation on positive affect.

Simple slope analyses revealed that for participants with a high orientation priority, a hedonic orientation was positively related with positive affect (*ß* = 0.22, 95% CI (0.06, 0.39), *p* = 0.009) (Figure 2). For participants with a medium or low orientation priority, the relationship between a hedonic orientation and positive affect were not significant (*ß* = 0.10, 95% CI (−0.05, 0.24), *p* = 0.177; *ß* = −0.02, 95% CI (−0.22, 0.17), *p* = 0.805).

Finally, we used negative affect as the dependent variable (Table 4). For the main effects in Step 1, negative affect was not related with a hedonic orientation (*ß* = 0.01, 95% CI (−0.13, 0.15), *p* = 0.894), a eudaimonic orientation (*ß* = −0.07, 95% CI (−0.22, 0.08), *p* = 0.369), or orientation priority (ß = −0.07, 95% CI (−0.22, 0.09), *p* = 0.383). In Step 2, negative affect was also not related with either the hedonic orientation × orientation priority interaction (*ß* = −0.05, 95% CI (−0.17, 0.07), *p* = 0.396) or the eudaimonic orientation × orientation priority interaction (*ß* = 0.02, 95% CI (−0.09, 0.13), *p* = 0.676).

## 4. Discussion

There has been an ongoing debate regarding what type of happiness orientation is most beneficial for individual well-being. Some scholars claim that individuals with high levels of hedonic and eudaimonic orientations simultaneously will obtain the highest level of happiness [3,4], whereas others suggest that pursuing hedonia does not always allow individuals to succeed in their aims and may sometimes even backfire [5,6]. The present study explored when hedonic and eudaimonic orientations were and were not positively related to individual well-being by investigating the potential moderating effects of orientation priorities.

Overall, a hedonic orientation was positively related with positive affect, but was not associated with psychological well-being. In contrast, a eudaimonic orientation was positively related with psychological well-being, but was not associated with positive affect. In addition, we found no evidence supporting an association between either hedonic or eudaimonic orientation and negative affect. These results are in accordance with some previous studies [3,39,40]. Combining previous findings with the current results, it can be suggested that hedonic and eudaimonic orientations are primarily related to different aspects of well-being.

Moreover, the current data supported our hypotheses that the relationships between hedonic and eudaimonic orientations and well-being are moderated by orientation priorities. The results revealed that if individuals prioritize eudaimonic goals over hedonic ones in situations with conflicting motives, and thus do not let the pursuit of hedonia interfere with the pursuit of eudaimonia, both hedonic and eudaimonic orientations could be beneficial for their well-being. In contrast, if individuals prioritize hedonic goals over eudaimonic ones, hedonic and eudaimonic orientations would not affect their well-being. However, inconsistently with our hypothesis, we did not find a negative association between hedonic orientation and individual well-being when the orientation priority was low. A possible reason is the small effect size, and thus a larger sample size may be required to detect this association [5].

Nevertheless, these findings shed some light on the conflicting findings on the relationship between orientations to happiness and well-being in previous research. It has been suggested that hedonia can be approached in either healthy or harmful ways, and excessive or unbalanced hedonic motives may lead to dysfunctional behaviors and may in turn be detrimental to individual well-being [41]. However, it is still unclear exactly what kind of hedonic orientation should be regarded as harmful. The current results suggest that one way to distinguish between unhealthy and healthy hedonic orientations is by its relative importance in relation to a eudaimonic orientation. Specifically, if individuals prioritize hedonic goals over eudaimonic ones, a strong hedonic orientation is likely to make them sacrifice long-term goals for momentary pleasure in situations where hedonic and eudaimonic motives are in conflict [13]. In this case, a hedonic orientation is likely to cause dysfunctional behaviors such as addiction and procrastination, and this can further impair well-being [7,28]. Moreover, although most previous studies found that a eudaimonic orientation had a stronger positive relation with individual well-being than a hedonic orientation, our findings suggested that the benefits of a eudaimonic orientation are not guaranteed. If people prioritize hedonia over eudaimonia, their eudaimonic motives stand less of a chance of being transformed into real action, thus contributing little to their well-being.

Interestingly, while orientation priority was used as a moderator in our theoretical model, our results showed that it also had main effects on individuals’ psychological well-being and positive affect. In other words, prioritizing eudaimonia over hedonia may boost happiness directly, perhaps by affecting individuals’ decision-making in situations with goal conflicts. One potential explanation for such strong direct effects is that situations with goal conflicts are more common than we expected. Pursuing eudaimonia in ways such as seeking personal growth or contributing to others typically requires individuals to exert extra efforts or make certain sacrifices [42,43], and is thus more or less in conflict with hedonic goals. Another reason may be that people’s behavioral choices in situations with goal conflict have a relatively stronger effect on their well-being. For instance, the well-being benefit brought about by having a party at an appropriate time may not cover the well-being lost by having a party which hampers important work the following day.

One issue which remains unclear is whether the present findings can be generalized to different cultures. On one hand, hedonic motives are more valued by individualistic cultures than collectivistic ones [44]. Thus, in collectivistic cultures such as China, people with strong hedonic motives may experience negative emotions (e.g., shame and guilt) due to conflict with cultural values and may encounter environmental constraints upon acting in a hedonic manner, especially when hedonic goals conflict with eudaimonic ones [5]. Supporting this view, Joshanloo and Jarden [10] found that hedonism had a stronger positive association with well-being in individualistic cultures. In this sense, orientation priorities may have less of an effect on people from individualistic cultures. On the other hand, even in individualistic cultures, the excessive pursuit of personal pleasure can lead to problematic behaviors and further impair well-being [7,41]. Consistently with this notion, Ford et al. [45] found that a motivation to pursue happiness was negatively related with well-being in individualistic cultures rather than in collectivistic cultures, because people from individualistic cultures seek happiness in less socially-engaged ways. In this case, it is reasonable to postulate that the moderating effect of orientation priorities would still exist in individualistic cultures. However, it is still too early to draw a conclusion. In future studies, it would be worthwhile to include culture-level factors (e.g., individualism vs. collectivism) and person-level factors (e.g., orientation priorities) simultaneously and investigate how they might interact to affect the relationship between the pursuit of hedonia and eudaimonia and individual well-being.

Some other limitations of the present study should also be noted. First, our sample comprised only undergraduate students. People in different stages of the lifespan have different hedonic and eudaimonic orientations [46]. It would be worthwhile to replicate our findings in different age groups. Another limitation of our study is that it was based on self-reported measures. Although the self-reported approach is the most commonly employed method in similar studies [1], it is susceptible to biases (e.g., social desirability). Thus, other assessment methods (e.g., peer reporting) should be considered in future studies to provide convergent evidence [47]. Finally, the mechanisms underlying the moderating effects of orientation priorities on the relationship between orientations to happiness and well-being require further clarification. Future research should consider including behavioral variables and investigating whether orientations to happiness and orientation priorities interact to affect both beneficial and harmful daily activities, thus further influencing well-being.

## 5. Conclusions

The present study contributes to an improved understanding of the relationships between orientations to happiness and well-being. In sum, hedonic and eudaimonic orientations are not always beneficial to well-being, and orientation priorities are a crucial factor that moderates these relationships. Hedonic and eudaimonic orientations contribute to improving individual well-being only when people prioritize eudaimonia over hedonia. In contrast, if people prioritize hedonic goals over eudaimonic ones, the positive effects of orientations to happiness on individual well-being will decrease or even disappear completely. These findings emphasize that orientation priorities, which have been much neglected in previous research, are at least of equal importance as the two orientations to happiness.

## Figures and Tables

**Table 1:** Means, standard deviations, and zero-order correlations of the study variables.

Variables	*M*	*SD*	1	2	3	4	5	6
1. Hedonic orientation	5.52	0.99	-					
2. Eudaimonic orientation	5.54	0.88	0.30 ***	-				
3. Orientation priority	0.36	0.94	−0.34 ***	0.48 ***	-			
4. Psychological well-being	5.28	0.74	0.06	0.32 ***	0.30 ***	-		
5. Positive affect	3.66	0.66	0.11	0.17 **	0.14 *	0.57 ***	-	
6. Negative affect	2.75	0.72	0.01	−0.10	−0.11	−0.42 ***	−0.50 ***	-

Note: *N* = 314. M = mean. SD = standard deviation. * *p* < 0.05, ** *p* < 0.01, *** *p* < 0.001.

**Table 2:** Regression results using psychological well-being as a dependent variable.

Model	*ß*	95% CI	*p*	*R* ^2^
Step 1				0.13
Hedonic orientation	0.10	(0.04, 0.23)	0.160	
Eudaimonic orientation	0.17	(0.03, 0.32)	0.018	
Orientation priority	0.25	(0.10, 0.39)	0.001	
Step 2				0.16
Hedonic orientation	0.05	(−0.09, 0.18)	0.486	
Eudaimonic orientation	0.23	(0.09, 0.38)	0.002	
Orientation priority	0.18	(0.03, 0.33)	0.022	
Hedonic orientation × Orientation priority	0.06	(−0.04, 0.17)	0.248	
Eudaimonic orientation × Orientation priority	0.15	(0.05, 0.26)	0.003	

Note: 95% CI = 95% confidence interval.

**Table 3:** Regression results using positive affect as a dependent variable.

Model	*ß*	95% CI	*p*	*R* ^2^
Step 1				0.05
Hedonic orientation	0.14	(0.00, 0.28)	0.046	
Eudaimonic orientation	0.05	(−0.10, 0.20)	0.483	
Orientation priority	0.16	(0.01, 0.31)	0.039	
Step 2				0.07
Hedonic orientation	0.10	(−0.05, 0.24)	0.177	
Eudaimonic orientation	0.10	(−0.05, 0.26)	0.196	
Orientation priority	0.10	(−0.06, 0.26)	0.234	
Hedonic orientation × Orientation priority	0.12	(0.01, 0.24)	0.033	
Eudaimonic orientation × Orientation priority	0.08	(−0.03, 0.18)	0.167	

Note: 95% CI = 95% confidence interval.

**Table 4:** Regression results using negative affect as a dependent variable.

Model	*ß*	95% CI	*p*	*R* ^2^
Step 1				0.01
Hedonic orientation	0.01	(−0.13, 0.15)	0.894	
Eudaimonic orientation	−0.07	(−0.22, 0.08)	0.369	
Orientation priority	−0.07	(−0.22, 0.09)	0.383	
Step 2				0.02
Hedonic orientation	0.02	(−0.13, 0.16)	0.831	
Eudaimonic orientation	−0.07	(−0.23, 0.09)	0.364	
Orientation priority	−0.06	(−0.22, 0.10)	0.465	
Hedonic orientation × Orientation priority	−0.05	(−0.17, 0.07)	0.396	
Eudaimonic orientation × Orientation priority	0.02	(−0.09, 0.13)	0.676	

Note: 95% CI = 95% confidence interval.

## Data Availability

Data supporting the reported results can be found at available online: https://osf.io/76mwx/?view_only=920ad181f6634e07a548db7e6a918473.
